# Effect of Thermal Processing on the Conformational and Digestive Properties of Myosin

**DOI:** 10.3390/foods12061249

**Published:** 2023-03-15

**Authors:** Miao Zhang, Shuran Zhu, Qian Li, Dejiang Xue, Shuai Jiang, Yu Han, Chunbao Li

**Affiliations:** 1 Key Laboratory of Meat Processing and Quality Control, Ministry of Education, Nanjing 210095, China; 2 Key Laboratory of Meat Processing, Ministry of Agriculture and Rural Affairs, Nanjing 210095, China; 3 Jiangsu Collaborative Innovation Center of Meat Production, Processing and Quality Control, College of Food Science and Technology, Nanjing Agricultural University, Nanjing 210095, China; 4 International Joint Collaborative Research Laboratory for Animal Health and Food Safety, Ministry of Education, College of Veterinary Medicine, Nanjing Agricultural University, Nanjing 210095, China

**Keywords:** myosin, structure, digestibility, pepsin, heating

## Abstract

Heat treatment affects the structural properties of meat proteins, which in turn leads to changes in their sensitivity to digestive enzymes, further affecting the nutritional value of meat and meat products. The mechanism of changes in the structure and digestive properties of myosin under different heating conditions were studied. An increase in heating temperature led to the exposure of internal groups to a polar environment, but to a decrease in the sturdy α-helix structure of myosin (*p* < 0.05). The results of tryptophan fluorescence verified that the tertiary structure of the protein seemed to be unfolded at 70 °C. Higher protein denaturation after overheating, as proven by the sulfhydryl contents and turbidity, caused irregular aggregate generation. The excessive heating mode of treatment at 100 °C for 30 min caused myosin to exhibit a lower degree of pepsin digestion, which increased the Michaelis constant (Km value) of pepsin during the digestion, but induced the production of new peptides with longer peptide sequences. This study elucidates the effects of cooking temperature on the conformation of myosin and the change in digestibility of pepsin treatment during heating.

## 1. Introduction

Heating is the most common method of meat processing, during which meat not only undergoes textural changes and develops flavor, but more importantly, pathogens are killed to ensure human consumption safety [1]. Different heating methods or degrees of heating can lead to different eating quality and consumer acceptance of the product [2]. Thus, researchers have been working on seeking suitable strategies to optimize cooking conditions for achieving satisfactory quality results (see review by Guo et al. [3]). Heating has been found to change the conformational structure of muscle proteins [4,5,6] which affects the digestibility of meat proteins [7]. Moreover, products of protein structural changes have adverse impacts on the texture or nutritive value of meat. It is therefore essential to control these unfavorable changes to further attract consumers.

Compared with the texture quality of processed meat products, the nutritional values of these foods are more focused on by modern consumers [7]. Admittedly, in vitro digestion has been widely regarded as one of the important indicators to evaluate the nutritional value of meat protein [8]. According to a previous report [9], meat proteins are initially digested by pepsin in the first step (stomach), which could be further degraded by other enzymes in the second part (e.g., trypsin in the small intestine). In contrast, the digestive process in the stomach is extremely essential for exploring whole meat protein digestibility as more meat protein fragments are reported in cooked meat [10]. Our previous study found that heating temperature has a more pronounced impact on the pepsin digestion of cooked pork compared with the pepsin−trypsin treatment [11]. Hence, it is necessary to investigate pepsin-treated digestive properties of protein when evaluating protein digestibility. Notably, pepsin-treated protein digestibility is closely related to structural characteristics [12].

Myosin accounts for about 50 % of the myofibrillar protein [13], which is considered to be the key part of determining the texture or nutritive value of meat products. Additionally, myosin is sensitive to the environment, which allows for the digestive properties of meat products. Therefore, myosin was selected as a pure protein for the simulation processing system. It should be noted that research on the impact of different heat procedures on the conformational and digestive properties of myosin contributed to the guidance of cooking the meat products.

This study was conducted to explore the effects of different heating procedures on the conformational and digestive properties of myosin. UV–VIS, intrinsic tryptophan fluorescence, and circular dichroism (CD) were applied to evaluate the structural changes of myosin after being heated at different parameters. Then, *sulfhydryl groups* and turbidity were also assessed to judge the protein aggregates under various conditions. The application of enzymatic reaction kinetics and LC-MS/MS was to explore the effect of different processing methods on the pepsin affinity of protein.

## 2. Materials and Methods

### 2.1. Materials and Reagents

The myosin (rabbit muscle, M1636), bovine serum albumin (BSA), and pepsin from porcine gastric mucosa (CAS No. 9001-75-6) were purchased from Sigma-Aldrich Chemical Co. (St. Louis, MO, USA). All other reagents (analytical grade) were obtained from Macklin Biochemical Co., Ltd. (Shanghai, China).

### 2.2. Preparation of Samples

The myosin was dissolved in 20 mM phosphate buffer (pH 7.0) containing 0.6 M NaCl, and the protein concentration was adjusted to 4 mg/mL for further use. Simulated cooking treatments were conducted via water bath equipment (TW20, Julabo, Seelbach, Germany) at 70 and 100 °C for 15 and 30 min, respectively. Then, the myosin after being heated was cooled through ice water.

### 2.3. UV–VIS Spectroscopy Measurement

The treated myosin solution was diluted to about 0.5 mg/mL through 20 mM phosphate buffer (pH 7.0) containing 0.6 M NaCl. Then, an evenly mixed myosin was added to a 96-well plate (200 μL) for the scanning of the UV–VIS spectra (270–300 nm) using a microplate reader (SpectraMax M2, Molecular Devices Ltd., Sunnyvale, CA, USA), and an average of three scans per spectrum were taken.

### 2.4. Fluorescence Spectroscopy Measurement

The fluorescence measurements of the myosin samples were conducted using a microplate reader (SpectraMax M2, Molecular Devices Ltd., Sunnyvale, CA, USA). Different myosin samples were diluted to 0.5 mg/mL to achieve better dispersion. Then, the excitation wavelength was set at 295 nm. The fluorescence emission spectra (300–400 nm) of myosin samples under different heating treatments were recorded in fluorescent curve form.

### 2.5. Circular Dichroism Measurement

The conformational changes of different myosin samples induced by heating were evaluated using a circular dichroism (CD) spectrometer (J-1500, Jasco, Tokyo, Japan). Different myosin samples were diluted to 0.05 mg/mL to achieve better dispersion. Then, the data of the CD spectra were collected through the method of Chen et al. [14], with some modifications. A quartz cuvette with a 0.1 cm path length was used. In addition, CD spectra were recorded from 200 to 250 nm based on a set procedure (scanning rate: 200 nm/min) at 25 °C.

### 2.6. Sulfhydryl Groups

The *sulfhydryl groups* of different myosin samples induced by heating were determined through absorbance (412 nm). Briefly, 100 μL of prepared myosin samples, 1 mL of Tris-glycine buffer (0.086 M Tris, 0.09 M glycine, 4 M EDTA, pH 7.0), and 20 μL Ellman’s reagent (10 mM DTNB in buffer, pH 7.0) were mixed in a water bath (25 °C, 1 h). The *sulfhydryl groups* were assessed using a UV−VIS spectrophotometer (U-3900, Hitachi Corp., Tokyo, Japan) at 412 nm. The *sulfhydryl groups* of the protein samples are be calculated as follows:

(1) $$Sulfhydryl\;groups\left(10^{-5}mol/g\right)=\frac{A\times c}{E_{m}}$$

where *A* is the measured absorbance, *c* is the myosin concentration, and *E_M_* is the molar extinction coefficient (13600).

### 2.7. Turbidity Measurement

The turbidity of different myosin samples was determined using a UV-vis spectrophotometer (U-3900, Hitachi Corp., Tokyo, Japan) at 340 nm. Firstly, different myosin samples were diluted to 1 mg/mL to achieve better dispersion. Then, an evenly mixed myosin was added to a 96-well plate (200 μL) for the scanning of turbidity.

### 2.8. Enzymatic Reaction Process

The pH values of the mixture containing 1 mL of the prepared myosin solution and pepsin solutions (49 mL) were adjusted to 2.0 through 0.1 M HCl. Then, these samples were moved to achieve an incubation at 37 °C for 2 h. In addition, 200 μL of the digestive solution was taken at 0–120 min for centrifugation (14,000× *g*, 10 min, 100 k ultrafiltration tube). The enzymatic reaction process was monitored at 280 nm using a NanoDrop spectrophotometer (NanoDrop Technologies, Rockland, DE, USA). In this study, the measured OD_280_ values were shown on the ordinates, whereas the heating time was exhibited on the abscissa.

### 2.9. Measurement of Pepsin’s Constant Michaelis (Km)

Different concentrations of myosin samples (0.3–0.7 mg/mL) were selected to be mixed with pepsin solution. Then, these samples were moved to achieve an incubation at 37 °C for 10 min. In addition, 200 μL of the digestive solution was taken at 0–10 min for centrifugation (Avanti JC, Beckman Coulter, CA, USA) (14,000× *g*, 10 min, 100 k ultrafiltration tube). The results were obtained at 280 nm using a NanoDrop spectrophotometer (NanoDrop Technologies, Rockland, DE, USA). In this study, the reciprocal of the reaction rate was shown on the ordinates, whereas the reciprocal of the substrate concentration was exhibited on the abscissa. Km could be directly achieved by the slope of the fitted curve.

### 2.10. Identification of Peptides in Digested Products

Different myosin samples were digested through the same procedure as described above. Then, the myosin samples were heated at 100 °C for 5 min to stop the enzymatic reaction. The digested products were identified based on the method of our previous study [8], with slight modifications. Briefly, the myosin mixtures were centrifuged at 14,000× *g* for 20 min to obtain the pure peptide. Subsequently, these peptides were separated by two different C18 columns (2 cm × 200 μm, 5 μm; 75 μm × 100 mm, 3 μm). In this sense, buffers A (0.1% formic acid, water) and B (0.1% formic acid, 84% acetonitrile) were used in the separation. Peptides were then identified through a hybrid quadrupole orbitrap mass spectrometer (Thermo Fisher Scientific, Waltham, MA, USA). Tolerance: 10 ppm, variable modification: Met oxidation. Proteome Discover-1.4 was applied to match the myosin with peptide data (http://www.uniprot.org/, accessed on 1 December 2022 ).

### 2.11. Statistical Analysis

All of the experiments were performed five times. The effects of heating time and temperature on the conformational and digestibility of myosin were evaluated using multiple ANOVA under the SAS program (version 9.2, 2009, SAS Institute Inc., Cary, NC, USA). For each variable, data were reported as means and standard deviations; means were compared by Ducan’s multiple comparisons and significant differences were considered between means if *p* values were smaller than 0.05.

## 3. Results and Discussion

### 3.1. Myosin Structural Changes during Heating

#### 3.1.1. UV–VIS Spectroscopy

**Figure 1:**
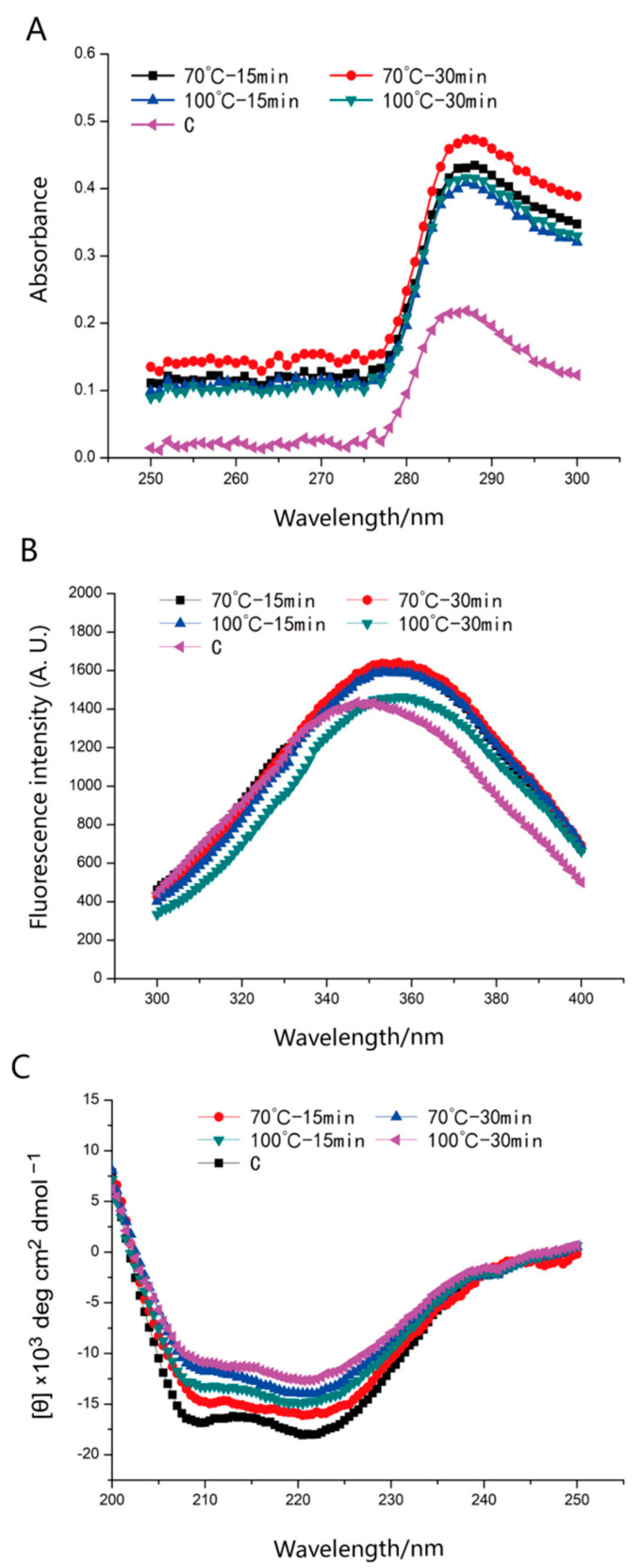
UV–VIS spectra (**A**), intrinsic tryptophan fluorescence (**B**), and CD spectra (**C**) of myosin after being heated at different cooking conditions.

UV–VIS spectroscopy is assumed to be a critical tool to detect the conformational transformations caused by the aggregation or unfolding process of myosin during heating [15]. Obvious adsorption peaks near 287 nm for all samples were shown in Figure 1A, corresponding to the UV adsorption of internal groups (e.g., tyrosine and tryptophan) of myosin. Compared with the control group, the intensity of the UV characteristic peak of all heat-treated groups increased, implying that heating exhibited excellent abilities in affecting the conformation of myosin. Moreover, no significant change in heat-treated groups (70 °C-15 min and 100 °C-15 min) was achieved in this study when the myosin was subjected to heating for 15 min. However, the heating treatment showed pronounced impacts on the intensity of maximum ultraviolet absorbance if the heating time was fixed for 30 min. In this regard, the UV absorption intensity of myosin after being heated at 70 °C was significantly higher (*p* < 0.05) than that obtained at 100 °C (Table 1). Interestingly, there were a positive correlation between heating time and the UV–VIS spectra intensity when the myosin was subjected to heating at 100 °C. The results indicated that the internal groups were gradually exposed to a polar environment during heating. Another study [8] also reported the unique effect of heating on protein conformation. In particular, overheating (100 °C) induced the aggregation of myosin, leading to the re-burial of some exposed amino acid residues. It corresponds to the decrease in the intensity of maximum ultraviolet absorbance (70 °C for 30 min and 100 °C for 30 min).

#### 3.1.2. Intrinsic Tryptophan Fluorescence

Protein tertiary structure variations can be monitored through intrinsic tryptophan fluorescence, which is often considered to be sensitive to the polarity changes of the myosin microenvironment [16]. In general, the complex spatial structure of myosin consists of a hydrophobic core stabilized by internal non-polar amino acids and packaged by external polar amino acids. The intrinsic tryptophan is easy to be transformed from the inner hydrophobic core to the polar environment when myosin is modified to unfold its tertiary structure [17]. Based on our results, the original tertiary structure of the myosin was remarkably changed during heating. As depicted in Figure 1B, the control group showed an apparent maximum emission peak (*λ*_max_) at 347 nm, while *λ*_max_ shifted to 357 nm after heating treatments were conducted. The noticeable red shifting of tryptophan fluorescence suggested the fact that the tryptophan fluorophore was brought to a more aqueous microenvironment, and the change degree was similar to that reported by Zhao et al., [18] who found a redshift in the PES-like chicken protein under extreme pH conditions. Another study [19] also concluded that myosin gradually appeared to be unfolded when the temperature rose from room temperature to 40 °C, consistent with a significant shift in fluorescence peak position.

In addition, mild heating (70 °C) promoted the denaturation of the protein, leading to a change in the position of tryptophan residues and consequently to its fluorescence emission [20]. Attenuated tryptophan fluorescence by overheating (100 °C, 30 min) was observed in myosin, implying that a relatively high heating temperature accelerated the aggregation of protein [21]. These results revealed the essential role of processing temperature in modifying the stable structure of myosin. The intrinsic tryptophan fluorescence results were consistent with those of the UV–VIS spectra, which indicated that excessive heating might destroy the natural impair structure of myosin.

#### 3.1.3. CD Spectra

The arrangement direction of peptide bonds determines the splitting of energy level transition, and the protein secondary structure is closely related to these properties [22]. Therefore, the variation in CD characteristic peaks can explain the secondary [14] structure of myosin well. CD measurements were conducted to evaluate the myosin secondary structure changes during heating. Two typical negative bands at 205–225 nm are displayed in Figure 1C, which were attributed to the α-helical structure [23] of the myosin tail. Broad peaks in the control group indicate that many initial myosin filaments were present before being heated, corresponding to an abundant α-helix fraction. Various heating treatments led to an obvious negative attenuation of these two peaks, implying significant disintegration or disruption of the compact structure of filamentous myosin.

When the heating time remained constant, the α-helix contents decreased apparently as the experimental temperature increased, and more significant reductions were observed at 100 °C (Table 2, *p* < 0.05). The ordered α-helix contents were decreased by 3.6% as the preset heating temperature was enhanced from 70 °C to 100 °C for 15 min, and by 12.9% for 30 min. In addition, the α-helix contents decreased apparently as the experimental time increased and more significant reductions were achieved at 30 min (*p* < 0.05) when the heating temperature remained constant. The original α-helix contents were decreased by 7.9% as the preset heating time was enhanced from 15 min to 30 min at 70 °C, and by 16.7% at 100 °C. Typically, the stability of α-helix structures is connected with intermolecular hydrogen bonds in myosin [24], which are buried in the interior sites of polypeptide chains. In this regard, the evaluation of the myosin secondary structure indicated that heating promoted a loss of the protein-dense helix structure, causing meat protein molecules to become more flexible and unordered than before. Li et al. [20] also reported a decrease in ordered α-helix proportion after microwave heating, which was in great agreement with our study. Additionally, the β-sheet components of heat-treated proteins were higher than those of the control group. The increase in β-sheet contents enhanced the interaction between protein molecules, which often contributed to the formation of myosin aggregates [25]. In this sense, a strong myosin interaction induced excessive protein aggregates, as confirmed by the largest β-sheet in the 100 °C for 30 min group.

#### 3.1.4. Reactive Sulfhydryl Content (RSC)

**Figure 2:**
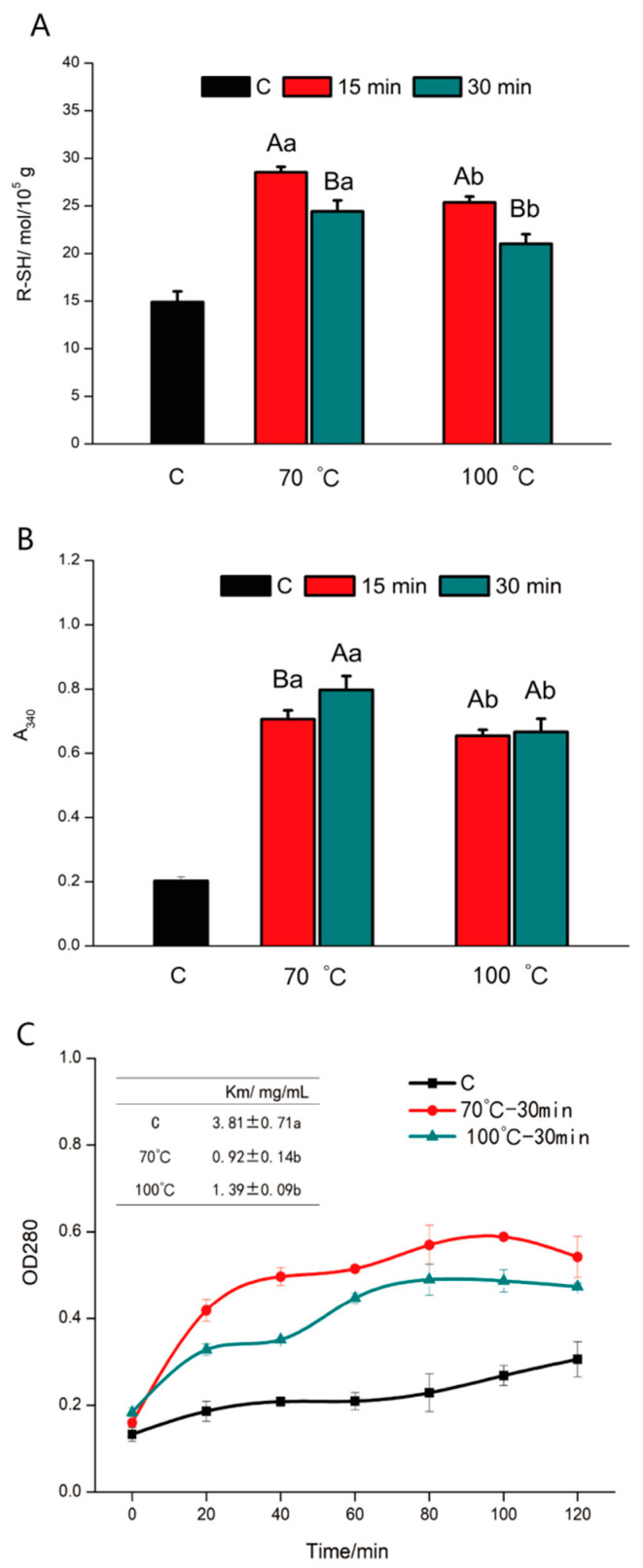
Sulfhydryl contents (**A**), turbidity (**B**), and enzymatic reaction process curve (**C**) of myosin after being heated at different cooking temperatures. Different capitals indicate a significant difference in heating time (*p* < 0.05); different small letters indicate a significant difference in heating temperature (*p* < 0.05).

RSC on the myosin surface has been reported [26] to be one of the most active groups in meat protein. In addition, the number of RSC in the myosin molecule is about 42, and most of them (about 68%) are present in the head of the myosin. For control and heat-treated samples (Figure 2A), the RSC of myosin increased with the progress of heating. Our data showed that myosin was unfolded during the change in cooking temperature for exposing more functional active groups. Notably, mild heating facilitated the enhancement of RSC. In comparison, overheating (100 °C) hindered the exposure of RSC, and this trend was not transformed by the change in heating time. The results show that the exposure of RSC in overheating (100 °C) system was not dominant. In other words, RSC gradually polymerized into disulfide bonds, consuming a certain amount of RSC. Xue et al. [27] also believed that excessive heating treatment (>70 °C) caused opposite RSC changes compared with the control protein, that is, the RSC in myosin complex enhanced apparently from 55 °C to 70 °C and then decreased slightly. As it has been reported earlier [28], myosin is sensitive to heat, which may cause the violent unfolding of the protein, impairing the exposure of functional active groups. More importantly, overheating (100 °C) is likely to destroy myosin detrimentally by forming high molecular weight polymers. As a consequence, the partially denatured meat proteins might interact with each other, forming irregular aggregates, and inhibiting the interaction between myosin and pepsin [29].

#### 3.1.5. Turbidity

Turbidity testing was often selected to evaluate the myosin aggregation or dispersion state during heating. Typically, a larger turbidity value means a higher degree of myosin aggregation. As the heating temperature expanded, the measured turbidity values increased (Figure 2B) for both the 70 °C and 100 °C-treated proteins. This could be ascribed to the fact myosin unfolded to expose more RSC during heating and thus myosin molecules aggregated via hydrogen bonds, which could interfere with light scattering, leading to an increase in the protein turbidity value. Another study reported a decrease in the protein turbidity value when the heating temperature increased above 50 °C. This non-monotonic trend of myosin turbidity during heating may be related to the multistep aggregation mechanism of the protein [30]. Although larger aggregation was formed after being heated at 70 °C, over a certain temperature, the structural advantage of heat-treated myosin may be reduced. In this sense, myosin forms irregular aggregates and settles [31], resulting in a decrease in turbidity. Therefore, we hypothesized that there may exist an essential heating temperature for heat-treated myosin to produce suitable meat protein products; an optimized temperature, over which, the detrimental enzymatic reaction may result from too much irregular myosin−myosin aggregation being formed after being heated. It confirmed the premise proposed above that overheating (100 °C) impaired the unfolding level of myosin and even resulted in irregular aggregation or dispersion.

Hence, the structural behavior of myosin was summarized as follows: myosin unfolded to expose active *sulfhydryl groups* after being heated, along with the destruction of α-helix and tertiary structure. Then, neighboring myosin interacted through hydrogen bonds for final structural rearrangement. In this regard, overheating (100 °C) led to the formation of irregular protein−protein aggregation through releasing excess energy. These structural properties may jointly determine the enzymatic reaction of pepsin.

### 3.2. Enzymatic Reaction

To monitor the enzymatic reactions of myosin during 120 min digestion of pepsin, the OD_280_ values were detected for the control group and the proteins heated at both 70 °C and 100 °C. The OD_280_ value of all myosin samples increased rapidly within 20 min of the initial enzymatic reaction (Figure 2C). This could be attributed to the presence of abundant active sites in meat proteins at the beginning of enzymatic hydrolysis, and this rapid hydrolysis was also reported by Zhao et al. [32]. The increasing rate of peptide production slowed down after enzymatic digestion for 80 min. In this situation, the enzymatic reaction process curve of myosin became smooth. Notably, the digestive rate of heat-treated myosin was significantly higher than that of the control group. Km value is one of the important constants reflecting the kinetic properties of the enzyme [33]. The hydrolysis rate of myosin by pepsin at 70 °C was faster than that at 100 °C, corresponding to a lower Km value (Figure 2C). The change in Km value implied that the binding affinity between myosin and pepsin increased after being heated [34]. In addition, mild heating exhibited the strongest binding affinity because of the moderate unfolding. Overheating (100 °C) increased irregular aggregates or the sediment of myosin and aggravated the resistance to pepsin action. This phenomenon was following the structure observations.

Digestive properties were mainly affected by heat-induced change degree of myosin. In particular, the utilization of the myosin active region by pepsin directly had an impact on the action of other proteolytic enzymes [35], thereby affecting the final bioavailability of the protein. Therefore, emphasizing the affinity between pepsin and myosin is an important step for investigating the digestive properties of the protein. Our previous study also summarized that enough force should be given to the pepsin-treated digestion process when an in vitro digestive system is selected to evaluate protein digestibility.

### 3.3. LC-MS/MS

**Figure 3:**
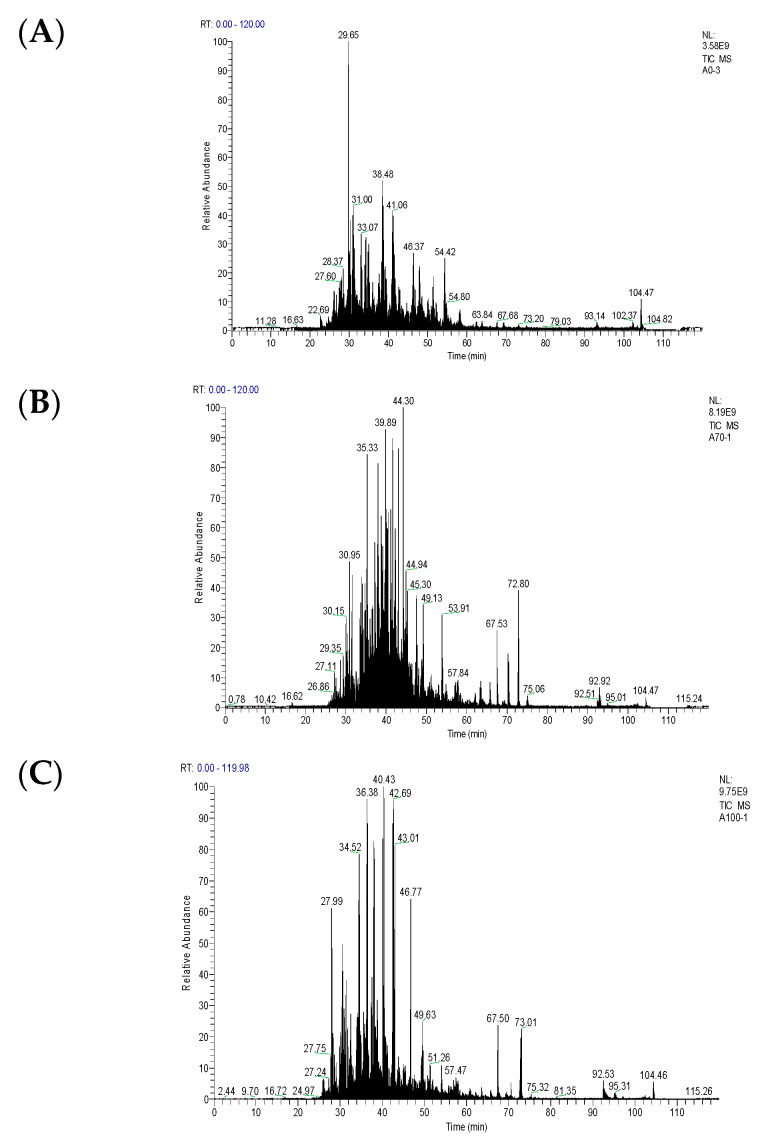
Representative total ion chromatogram spectra of in vitro digested products of myosin with different heating degrees ((**A**): Control, (**B**): 70 °C-30 min, (**C**): 100 °C-30 min).

The digestive products of myosin after being heated at different degrees were identified by LC-MS/MS [36]. In the present study, various heating treatments altered the distribution of peptide coming from pepsin digestion, especially at 70 °C (Figure 3). It should be noted that the abundance of myosin peptide in the range of 300–1800 m/z increased after pepsin digestion. This was consistent with the presentation of the enzymatic process curve and Km value, indicating that the affinity between myosin and pepsin increased after being heated. In addition, unique peptide sequences are shown in Table 3. A total of 4 shared peptides were identified in the pepsin digestion products of myosin in the control, mild heating (70 °C for 30 min), and overheating (100 °C for 30 min) group. The specific peptides for these three groups were 1, 2, and 3, respectively. The identified peptide sequence (ITKQEYDEAGPSIVHRKCF) in the control and mild heating group disappeared as the heating temperature increased from 70 °C to 100 °C for 30 min. In contrast, a brand new peptide with a longer peptide sequence (GEAAPYLRKSEKERIEAQNKPFDAKSSVF) was found. These results confirmed the previous findings of the kinetics results of the enzymatic reaction. Zhao et al. [37] concluded that heating at a higher temperature (100 °C) damaged partial cleavage sites for the digestive proteases. More so, the number and type of peptide sequences differed with muscle type [38].

Here, the change in myosin digestibility can be simply ascribed to two different aspects. First, the conformational changes of myosin during thermal treatment, especially the myosin tail unfolding and tertiary structure change, may affect the reaction process of the enzyme. The other is the irregular aggregates of myosin after being heated at 100 °C. In the system, neighboring myosin interacted through noncovalent forces for producing irregular protein−protein aggregation. Furthermore, these protein aggregations can also be generated by excessive protein oxidation during heating. In other words, irregular myosin aggregation has a certain degree of tolerance towards the action of pepsin.

At present, most meat products need to be heated before they can be eaten. Of all the meat proteins, myosin accounts for about 25% of meat and is the main component of myofibrillar protein (~50%), which affects the texture quality of meat and plays a critical role in digestive properties. In this study, the *sulfhydryl groups* in the myosin molecule were gradually exposed and then transformed into disulfide bonds after different thermal treatments. In this context, protein oxidation was also enhanced during heating [39]. The above results also suggest that the disorder degree in the secondary structure of myosin was enhanced by increasing heating temperature, corresponding to the reduction of the α-helix structure. Studies reported by Ding et al. [40] showed that the ATPase activity of the myosin head decreased and the molecular structure of its rod tail extended after being heated. Liu et al. [41] found that thermal treatment induced the conversion of pork myosin α-helix to β-sheet and random coil, followed by protein aggregation in the form of a spatial network morphology. Additionally, the tertiary structure of the myosin unfolded, resulting in the gradual exposure of internal active groups. Notably, non-covalent interactions between functional groups may cause misfolding and irregular aggregation between myosin molecules. Taken together, mild thermal treatment (70 °C) promoted the orderly unfolding of myosin, while overheating (100 °C) intensified the generation of polymers stabilized by both covalent (disulfide bonds) and non-covalent forces (hydrogen bonds), thereby affecting the enzymatic binding ability of myosin.

Heat-induced conformational changes in meat proteins are critical for increasing myosin digestibility. Wen et al. [11] reported that the increased exposure of protease cleavage sites after being heated at 70 °C was conducive to the digestion by pepsin when they studied the impacts of cooking temperature on the digestive properties of pork protein. However, abundant peptides from pepsin-digested proteins under relatively lower heating degrees disappeared as the heating temperature enhanced at about 100 °C. Escudero et al. [42] also concluded that peptides after thermal treatment were rich in proline residues, thereby letting them more resistant to digestive enzymes. Furthermore, the transformation of myosin spatial structure had a great influence on protein digestion in the gastric part due to the shear specificity of pepsin. In the current study, the exposure of myosin tryptophan residues and *sulfhydryl groups* in the mild heating group was greater than those in the overheating group. However, more obvious secondary structure and protein aggregation changes were shown in the overheating group. In this sense, mild heating induced myosin to expose more cleavage sites in contact with pepsin, leading to a lower Km value. Therefore, protein tertiary structure and *sulfhydryl groups*, rather than the secondary structure, should be firstly taken into consideration when studying the digestive properties of myosin during heating.

## 4. Conclusions

With the increase in heating temperature, the conformational properties (UV–VIS spectra, intrinsic tryptophan fluorescence, secondary structure, RSC, and turbidity) of myosin were altered. Myosin was unfolded to expose active *sulfhydryl groups* after being heated, along with the destruction of α-helix and tertiary structure. Then, neighboring myosin interacted through hydrogen bonds for finally structural rearrangement. Structure changes during mild heating (70 °C) induced the exposure of more active sites for pepsin. However, overheating (100 °C) caused a relatively reverse result. LC-MS/MS showed that overheating induced the production of a new peptide with a longer peptide sequence, which may be related to the increase in irregular aggregates of myosin after being heated at 100 °C. Our study indicates that a moderate cooking method could improve pepsin sensitivity and further studies should be conducted on its nutrition.

## Figures and Tables

**Table 1:** The effects of different heating treatments on the characteristic peaks of UV−VIS spectroscopy (x ± s, *n* = 5).

	Abs_287 nm_
Unheated	0.22 ± 0.01
70 °C-15 min	0.43 ± 0.02 ^Ba^
70 °C-30 min	0.47 ± 0.02 ^Aa^
100 °C-15 min	0.41 ± 0.02 ^Aa^
100 °C-30 min	0.42 ± 0.02 ^Ab^

Note: Different capital letters indicate a significant difference in heating time (*p* < 0.05); different small letters indicate a significant difference in heating temperature (*p* < 0.05).

**Table 2:** Content of secondary structure units of myosin under different heating degrees (x ± s, *n* = 5).

	70 °C		100 °C		Unheated
	15 min	30 min	15 min	30 min	
α-helix	30.86 ± 0.49 ^Aa^	28.42 ± 1.34 ^Ba^	29.74 ± 2.13 ^Aa^	24.76 ± 3.13 ^Bb^	31.44 ± 1.15
β-sheet	0.00 ± 0.00 ^Aa^	1.00 ± 2.23 ^Ab^	0.60 ± 0.83 ^Ba^	22.08 ± 5.39 ^Aa^	0.00 ± 0.00
β-turn	32.78 ± 0.32 ^Bb^	36.46 ± 2.33 ^Aa^	35.80 ± 0.57 ^Aa^	28.40 ± 6.03 ^Bb^	33.62 ± 1.60
Randow coil	36.38 ± 0.65 ^Aa^	34.14 ± 1.43 ^Aa^	33.86 ± 2.14 ^Aa^	29.14 ± 2.41 ^Bb^	34.94 ± 2.40

Note: Different capitals indicate a significant difference in heating time (*p* < 0.05); different small letters indicate a significant difference in heating temperature (*p* < 0.05).

**Table 3:** The digested peptides of myosin with different heating degrees.

Peptide Sequence	Control	70 °C	100 °C
RQRYRILNPAAIPEGQF	0	1	0
ITKQEYDEAGPSIVHRKCF	1	1	0
LMKILTERGYSF	0	1	1
IGMESAGIHETTY	1	1	1
MNVKHWPW	1	0	0
SLVHYAGTVDY	1	1	1
TVIDQNRDGIIDKEDLRDTF	0	0	1
MNVKHWPWMKLYF	0	1	0
DDMEKIWHHTFY	1	1	1
GEAAPYLRKSEKERIEAQNKPFDAKSSVF	0	0	1
DDMEKIWHHTF	0	0	1
TNEKLQQFFNHHMF	1	1	1
TITDLPTDAKIF	0	1	1

Note: 0 indicates that the peptide did not appear, and 1 indicates that the peptide appeared.

## Data Availability

All related data and methods are presented in this paper. Additional inquiries should be addressed to the corresponding author.
